# Inflammatory Bowel Disease: A Potential Result from the Collusion between Gut Microbiota and Mucosal Immune System

**DOI:** 10.3390/microorganisms7100440

**Published:** 2019-10-11

**Authors:** Bei Yue, Xiaoping Luo, Zhilun Yu, Sridhar Mani, Zhengtao Wang, Wei Dou

**Affiliations:** 1 Shanghai Key Laboratory of Formulated Chinese Medicines, Institute of Chinese Materia Medica, Shanghai University of Traditional Chinese Medicine (SHUTCM), Shanghai 201203, China; YBrunning@163.com (B.Y.); luoxpcq@163.com (X.L.); yuzhilunabc@163.com (Z.Y.); 2 Departments of Medicine and Genetics, Albert Einstein College of Medicine, The Bronx, NY 10461, USA; sridhar.mani@einstein.yu.edu

**Keywords:** Inflammatory bowel disease, mucosal immune system, gut microbiota

## Abstract

Host health depends on the intestinal homeostasis between the innate/adaptive immune system and the microbiome. Numerous studies suggest that gut microbiota are constantly monitored by the host mucosal immune system, and any slight disturbance in the microbial communities may contribute to intestinal immune disruption and increased susceptibility to inflammatory bowel disease (IBD), a chronic relapsing inflammatory condition of the gastrointestinal tract. Therefore, maintaining intestinal immune homeostasis between microbiota composition and the mucosal immune system is an effective approach to prevent and control IBD. The overall theme of this review is to summarize the research concerning the pathogenesis of IBD, with particular focus on the factors of gut microbiota-mucosal immune interactions in IBD. This is a comprehensive and in-depth report of the crosstalk between gut microbiota and the mucosal immune system in IBD pathogenesis, which may provide insight into the further evaluation of the therapeutic strategies for IBD.

## 1. Introduction

The exact pathogenesis of inflammatory bowel disease (IBD) is still elusive, but it is generally accepted that the inflammation results from a defective mucosal immune response to intestinal flora in genetically susceptible individuals [1]. A common type of IBD is Crohn’s disease (CD), in which inflammation is usually transmural and can be found in any area of the gastrointestinal tract. Another major type of IBD, ulcerative colitis (UC), is characterized by a non-transmural inflammation that usually affects the colon and rectum [2]. The highest occurrence of IBD is in developed countries, such as those in North America and Europe, affecting up to 0.5% of the general population [3].

Since urbanization and rapid industrialization in developing countries, traditional lifestyles have changed greatly [4]. The clear relationship between the lifestyle changes associated with industrialization and the incidence of IBD has prompted exploration into the pathogenesis of IBD [2]. Lifestyle changes during urbanization, including improved sanitation, reduced early life microbial exposure, westernized diet, and increased antibiotic use, have been shown to influence the gut microbiota [5]. Furthermore, several lines of evidence support the hypothesis that disturbance of the relationship between the gut microbiota and the mucosal immune system is involved in IBD pathogenesis [6,7,8].

The mammalian gut is colonized by a large number of microorganisms, including bacteria, fungi, viruses, protists, and helminths, which are collectively called the gut microbiota or the microbiome [9,10,11]. Microbes take part in many physiological host processes, such as the biosynthesis of certain bioactive secondary metabolites. Furthermore, the microbiota plays an important role in maintaining the normal intestinal epithelial barrier, immune homeostasis, optimal immune responses, and protection against pathogen colonization [12].

Although most of the gut microbiota are mutualistic or commensal, when “dysbiosis” occurs under certain circumstances, pathogenic bacterial overgrowth can induce certain inflammatory diseases, such as IBD [13]. In this review, we will discuss the collusion between the gut microbiota and the mucosal immune system during the development of IBD.

## 2. Microbiota Dysbiosis as a Potential Trigger for IBD

### 2.1. Specific Pathogenic Microbes in IBD

The gut lumen has a large mucosal interface (300–400 m^2^) that has structures and functions related to immunological recognition of the xenobiotics from the environment [14]. The result of early research suggested that specific pathogenic microbes caused IBD, because many infectious pathogens result in diarrhea and lead to intestinal mucosal inflammation, similar to IBD [15]. *Mycobacterium avium* subspecies *paratuberculosis* is one such pathogen, and it has been widely studied for its potential role in the pathogenesis of CD [16,17]. Although the association of pathogenic microbes with CD seems to be specific, further studies on its regulation in the etiology of CD remain to be defined [18,19].

Another pathogenic microorganism attracting research interest is adherent-invasive *Escherichia coli* (AIEC). There is growing evidence that AIEC may contribute to the pathogenesis of IBD, especially CD [20]. Compared with healthy subjects, the AIEC richness index in CD patients is significantly increased, and a study has shown that the AIEC protease Vat-AIEC can contribute to intestinal mucosal injury and bacterial colonization [21]. Defensins secreted by Paneth cells play an important role in intestinal mucosal immunity, and intestinal mucosal cell surfaces with high concentrations of defensin also have high AIEC concentrations, suggesting that AIEC might have developed resistance to defensins [22]. A new strain of AIEC, LF82, has been shown to enter and survive in lamina propria macrophages and intestinal epithelial cells (IECs), followed by nuclear factor (NF)-κB signaling activation and TNFα secretion [23]. A recent study by Viladomiu et al. found that interleukin (IL)-17^+^ CD4^+^ T cells and RORγt^+^ CD4^+^ T cells were increased in both the colonic and small intestinal lamina propria after AIEC 2A colonization of germ-free C57BL/6 mice [24]. This indicates that AIEC 2A can increase Th17 polarization and effect mucosal immunity. All in all, a large amount of evidence shows that AIEC may contribute to the development of CD, while the signaling pathways involved in intestinal mucosal immunity remain less clear.

### 2.2. Profiles of the Intestinal Bacteria and IBD

In recent years, with the development and application of high-throughput sequencing, new techniques (e.g., 16S ribosomal RNA genes sequencing) have provided new approaches for exploring the effect of the gut microbiota in the pathogenesis of IBD [15,25]. Studies have been able to explore the whole bacterial community structure rather than a single or a few bacterial species. An increasing body of evidence suggests that neither a single nor a few pathogenic bacteria, but rather the change in the whole bacterial community structure, may cause IBD [26,27]. Research based on 16S rDNA sequencing has highlighted that only 7–9 of the 55 known bacterial divisions or phyla are detected in human fecal or gut mucosal samples [11]. *Bacteroidetes* (16%–23%) and *Firmicutes* (49%–76%) are the most abundant human gut bacteria, and less abundant phyla include *Proteobacteria*, *Fusobacteria*, *Actinobacteria*, and *Verrucomicrobia* [14,28,29]. Co-evolutionary relationships have been found between the host and symbiotic bacteria (including commensals and mutualists) [30]. Changes in host age, diet, or antibiotic use can cause a shift in symbiotic bacteria. In a healthy human body, after a temporary shift, the fecal bacteria have a tendency to return to its typical original structure [31]. The bacterial component of the microbiota provides considerable benefits to the host by generating metabolites, promoting the development of the mucosal immune system, and preventing colonization by pathogenic microorganisms [32]. However, after developing IBD, intestinal micro dysbiosis (imbalance between protective and harmful bacteria) is often found [27]. A widely recognized hypothesis is that intestinal micro dysbiosis can be a trigger for IBD [27].

Intestinal micro dysbiosis has been extensively described in patients with IBD. For example, reduction in diversity, changes in composition (increased or decreased abundance of specific species), and changes in metabolites occur [14,33,34]. Regarding the reduction in diversity, mucosal biopsies from twin pairs (including dizygotic and monozygotic twins) with UC have shown a reduction in gut microbiota diversity in both siblings relative to healthy individuals, indicating a reduction in the diversity of gut microbiota may contribute to IBD [35]. Additionally, studies of bacteria from UC patients also showed a lower fecal bacterial diversity than healthy individuals [36]. Moreover, in the first two years of life, lower diversity of bacteria in the gut is related to a reduction in T helper 1 (T_H_ 1) responses, which may contribute to the development of IBD in adulthood [37]. Regarding the changes in composition, many studies have shown that the gut microbiota in IBD patient exhibits increased *Proteobacteria* and reduced *Firmicutes* [38,39,40]. Moreover, decreased abundance of Clostridium cluster IV (the *Clostridium leptum* group), especially *Faecalibacterium prausnitzii*, has been reported [40,41]. Regarding the changes in metabolites, short-chain fatty acids (SCFA) formed by gut microbiota after the digestion of various dietary fibers can be absorbed and utilized by IECs [34]. A total of 95% of SCFAs may be allocated to their rapid absorption, with only about 5% being passed out of the body in the feces. Acetate, propionate, and butyrate are the main components in SCFAs, of which acetate can be produced through the Wood–Ljungdahl pathway by *Blautia hydrogenotrophica*, and propionate is generated by *Bacteroidetes* and *Firmicutes* through the succinate and lactate pathway, and the remaining butyrate being produced by several *Firmicutes* through Acetyl-CoA [42]. It has long been known that European children, who are more susceptible to IBD, have worse fiber digestive capability and lower SCFA levels than African children [43]. On the other hand, other evidence shows that SCFAs involve in regulating immunity and controlling inflammation, suggesting the role of SCFAs in maintaining intestinal homeostasis [34].

Nevertheless, studies on mucosal biopsies from IBD patients have revealed an increase in members of the *Enterobacteriaceae* family and a decrease in members of the *Clostridiales* order [43]. In colonic specimens, bacteria (including bacteria of the gamma subdivision of *Proteobacteria*) were found to invade the mucosa in 83% and 25% of UC and CD patients, respectively, compared with 0% of the controls without IBD [44,45]. In vitro experiments have indicated that several strains of bacteria from CD or UC patients, including *E. coli*, *Enterococcus faecalis*, and *Fusobacterium varium*, can erode IECs [46,47,48]. However, emerging technologies (e.g., DNA sequencing technologies and computational tools) have also drawn researchers’ attention to other gut microbes, such as fungi and viruses [49].

### 2.3. Fungal Microbiota and IBD

Whole-genome sequencing analysis indicates that >99% of the gut microbiota is bacteria, while fungi only account for 0.1% [29]. However, fungi have been suspected to be involved in the pathogenesis of IBD for a long time. Many years earlier, researchers regarded anti-*Saccharomyces cerevisiae* antibodies (ASCA) as a kind of serological biomarker for CD, indicating an excessive immune response to fungi in CD patients [50,51]. Furthermore, ASCA can be detected in 50%–60% of CD patients compared with only 8%–20% of healthy subjects [52]. Recent studies show that alterations in the fungal community composition and structure also exist between IBD patients and healthy subjects. IBD patients have a decreased *Ascomycota/Basidiomycota* ratio compared with healthy individuals, which involves an increased abundance of *Candida albicans* and a decreased abundance of *Saccharomyces cerevisiae* in IBD patients [53]. Fungal diversity is also dramatically reduced in IBD [53]. Moreover, in a mouse model of colitis, *C. albicans* aggravated intestinal inflammation while *S. cerevisiae* decreased inflammation [54,55]. Treatment of mice with an antifungal agent increased susceptibility to acute and chronic colitis [56]. Furthermore, the fungal community in the mammalian gut can interact with the immune system via the innate immune receptor Dectin-1 and the Card9-Syk signaling axis, maintaining intestinal homeostasis [53,54]. These findings provide objective evidence that the fungal “mycobiota” regulate the immune system and impact the incidence of IBD.

### 2.4. Enteric Virome and IBD

The intestine contains a large and complex viral community, which is known as “the enteric virome” [57]. The development of metagenomics has helped researchers to reveal the diverse composition of the enteric virome, which contains eukaryotic viruses (e.g., herpesviruses, adenoviruses, and uncharacterized eukaryotic viruses) and prokaryotic viruses (e.g., *Microviridae* and *Caudovirales*) [58,59]. However, so far, little is known about the role of the enteric virome in IBD. Recent animal studies have indicated that the enteric virome is involved in the pathogenesis of IBD. A eukaryotic virus, murine norovirus (MNV), disrupted gut homeostasis in IBD-susceptible mice (IL10^-/-^ and Atg16L^-/-^ mice) and induced serious colitis [60,61]. However, a model of bacteriophage adherence to mucus indicated that there is a symbiotic relationship between bacteriophages and the intestinal mucosa, that is, the mucus provides a habitat for bacteriophages, which provide defense against other microbes [62]. Moreover, a study of MNV infection of germ-free or antibiotic-treated mice found that MNV contributed to restoring the normal intestinal morphology and maintaining the innate immune functions [63]. Furthermore, research on the enteric virome in healthy subjects suggests that bacteriophages comprise much of the virome, and the species are relatively stable [64,65]. *Microviridae* and *Caudovirales*, which latently infect their bacterial hosts and generate offspring, representing the main dominant bacteriophage species [66,67]. However, alterations in bacteriophage species composition, also known as dysbiosis of the enteric virome (that is, increased levels of bacteriophages, particularly *Caudovirales*) have been found in IBD patients. Most interestingly, there is a predator–prey relationship between bacteriophages and their bacterial hosts, which is called a “transkingdom interaction,” and which may contribute to disease pathogenesis [11,57]. In summary, enteric virome affects the mucosal immunity at least in some respects, but its relationship to intestinal homeostasis remains to be investigated.

### 2.5. Protozoans and IBD

Although it is clear that the dysbiosis of bacteria, fungi, and viruses can impact intestinal homeostasis, the potential homeostasis-maintaining role of other microbial kingdoms, such as Protista, has seldom been studied. It is generally known that intestinal pathogenic protozoans, which are unicellular eukaryotes including *Cryptosporidium* spp., *Giardia* spp., *Entamoeba histolytica*, *Encephalitozoon cuniculi*, and *Toxoplasma gondii*, can cause diseases in mice and humans [68,69,70,71,72]. Furthermore, it was traditionally believed that any protozoan in human intestines was a parasite that could cause pathogenicity in the host body [73]. However, interestingly, emerging evidence suggests that some common protozoa inhabiting the human intestines are beneficial rather than harmful [74]. A growing body of research demonstrates that intestinal protozoans, such as *Blastocystis* and *Dientamoeba fragilis*, are also found at high levels in healthy individuals [75]. Many other symbiotic protozoans (e.g., *Entamoeba dispar* and *Pentatrichomonas*) are also present in the intestines [76].

There has been little research on the effects of intestinal protozoans on the development of IBD, especially regarding their effects on intestinal mucosal immunity. *Tritrichomonas musculis* (*T.mu*), a commensal intestinal protozoan of rodents, colonizes the bowel lumen and leads to inflammasome activation in epithelial cells and IL-18 and IL-1β release. *T.mu*-driven IL-18 can protect the intestinal mucosa against bacterial invasion but also promote the development of chronic colitis in mice [77]. Another *Tritrichomonas* species, *Tritrichomonas muris*, can dramatically increase the abundance of intestinal tuft cells (critical sentinels in the intestinal epithelium) and then affect type 2 innate lymphoid cells (ILC2s) via Trpm5 and the expression of cytokines, such as IL-25 and IL-13 [78]. Notably, a new hypothesis is that intestinal pathogenic protozoans need certain stimulatory factors (e.g., transkingdom interactions with certain intestinal bacteria) to activate their pathogenicity [79]. However, the protozoa, whether pathogens or commensal remains perplexing. There is no clear evidence that certain protozoans are useful to support intestinal health. The potential benefits of intestinal protozoans may be derived from increased intestinal biodiversity or their ability to regulate the host intestinal mucosal immunity.

### 2.6. Helminths and IBD

The “IBD hygiene hypothesis” proposed that bringing up children in extremely sanitary environments (e.g., with lower exposure to helminths) adversely affects the construction of the innate immune system, which contributes to susceptibility to IBD in later life [80]. Many recent clinical studies have demonstrated that various helminths (e.g., *Trichuris trichiura*, *Trichuris suis*, and *Necator americanus*) can alleviate IBD symptoms, and their absence has been associated with the development of IBD [5,81]. The most likely underlying mechanism is that helminths can alter immune responses (depress or decrease the release of inflammatory factors) in their hosts by releasing various excretory–secretory (ES) products [82]. Research on the effects of helminth infection utilizing an IBD-susceptible mouse model (nucleotide-binding oligomerization domain-containing protein 2 [Nod2]-knockout mice) has shown that parasitic *Trichuris muris* can ameliorate abnormal intestinal barriers (increasing the quality of goblet cells) and alter the balance of commensal and pathogenic bacteria [83,84]. Moreover, various helminths, such as *Echinococcus granulosus, Trichinella spiralis, Heligmosomoides polygyrus*, and *Ancylostoma caninum*, have been shown to protect against colitis in animal models [81]. Additionally, Sj16, a secreted protein of *Schistosoma japonicum*, has immunoregulatory protective effects on dextran sulfate sodium (DSS)-induced colitis by inhibiting the peroxisome-proliferator activated receptor-alpha (PPAR-α) signaling pathway, increasing Treg percentages and up-regulating anti-inflammatory factors production [81]. As mentioned above, intestinal helminth infection may protect against IBD through the regulation of multiple immune responses.

## 3. Mucosal Immune System and Intestinal Homeostasis

### 3.1. Composition of the Mucosal Immune System

The intestinal mucosal immune system comprises three barriers against harmful factors and maintains intestinal homeostasis [85]. The mucus layer covering the epithelial surfaces of the intestinal lumen is the first barrier. This barrier is composed of a complex polymeric network of highly glycosylated mucins (MUC proteins), which keeps microorganisms away from the IECs [86]. The second barrier is the single layer of IECs organized in intestinal structures, which is composed of multiple cell types, including goblet, enteroendocrine, tuft, columnar epithelial, and M cells. The third barrier is the numerous immune cells residing in the gut or scattered throughout the gut epithelium and lamina propria, including the mesenteric lymph nodes and Peyer’s patches [85]. All three barriers are important for preventing commensal microorganisms’ access to the systemic circulation and maintaining intestinal homeostasis, and any damage or functional abnormality of these barriers may cause CD and UC [87,88].

### 3.2. Mucus Layer

The mucus of the large intestine is largely produced and secreted by goblet cells; this mucus layer forms a complex network to produce a physical and biochemical barrier in the colon [89]. This barrier includes two layers, the inner and outer mucus layer. In a healthy gut, the inner mucus layer is impregnable to any commensal microorganisms. However, the outer mucus layer is more exposed to the intestinal lumen and provides a habitat for commensal microorganisms [90]. Mucus mainly comprises mucin glycoproteins, but it also acts as a medium for retaining other proteins, such as antimicrobial peptides (AMPs) and secretory immunoglobulin A (SIgA). Mucin 2 (MUC2) is a kind of gel-forming mucin that is most highly expressed in the colon, forming a stable well-organized structure that is almost completely free from bacteria [91]. SIgA, which is secreted across the IECs by plasma cells, is the main antibody of mucosal immunity and binds to pathogens to prevent their direct interaction with the host [92].

In a healthy human, the colonic epithelium is covered by the mucus, but in IBD patients, the percentage of the epithelium covered by mucus is significantly decreased, and the mucus is thinner and damaged [93]. An integrated mucus layer ensures that there is no direct contact between pathogens and IECs [93]. In contrast, in several genetic and chemically induced mouse models of colitis, pathogens are close to or even invade the IECs [94]. Moreover, tests on colonic specimens proved that mucosal bacterial invasion is common in IBD patients, while no invasion occurs in healthy controls [49].

### 3.3. Single Layer of IECs

The main structure of the intestinal barrier is formed by IECs, which not only create a physical barrier between symbiotic and pathogenic microbes and the lamina propria, but also play a prominent role in intestinal immunity against pathogenic bacteria and their components (e.g., lipopolysaccharides, LPS) [95]. Tight junction formation, mucus, and AMP secretion are examples of the immune function of IECs. IECs can be divided into absorptive cells (columnar epithelial cells) and secretory cells (goblet, enteroendocrine, and tuft cells) according to their biological functions [96]. Columnar epithelial cells, which are responsible for absorbing digested nutrients, are the main absorptive enterocytes in the intestinal epithelium [97]. Goblet cells, an indispensable secretory-type IECs, can synthesize and secrete gel-forming mucin, especially MUC2 [96]. Enteroendocrine cells, which represent about 1% of IECs, can release gut hormones to control gut movement and regulate food intake [98]. Tuft cells tend to be found later than other types of IECs, and they account for about 0.4% of IECs. Recently, it has been found that tuft cells act as critical guards in the intestinal mucosal immune system, promoting the recognition of and immunity against intestinal parasites [78,99]. Thus, IECs play a crucial role in maintaining intestinal homeostasis and participating in commensal–host interactions.

### 3.4. Intestinal Immune Cells

**Figure 1:**
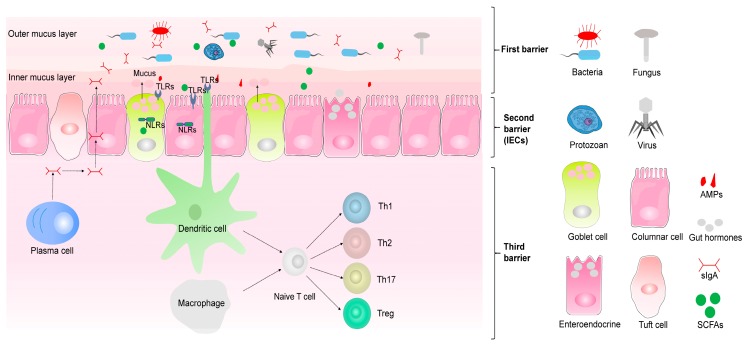
Cross-kingdom biological transmission and communication maintain intestinal homeostasis. Intestinal homeostasis is maintained by three immunological barriers: mucus layer (first barrier), epithelium layer (second barrier), and immune cell layer (third barrier). The mucus layer contains multiple immune mediators such as antimicrobial peptides (AMPs) and secretory immunoglobulin A (SIgA), which limit direct contact between the millions of microorganisms (including bacteria, fungi, viruses, and protists) and the intestinal epithelial cells (IECs). However, microorganisms are responsible for the degradation and digestion of dietary fiber to produce high-energy materials (e.g., short-chain fatty acids [SCFAs]) for the IECs. The IEC layer, which contains multiple pattern recognition receptors (PRRs), such as toll-like receptors (TLRs) and nod-like receptors (NLRs), is the second immunological barrier. It rapidly detects and responds to bacteria that invade the intestinal tissue. Finally, the immune cell layer promotes the monitoring and clearance function of the IECs to limit the access of enteric microbes, thus ensuring that “unlucky” invaders are killed rapidly while also promoting intestinal homeostasis.

There are many kinds and a large number of immune cells in the intestines, which play key roles in maintaining intestinal homeostasis. Changes in their morphology and functions may lead to IBD [100]. Recently, a growing number of studies have begun to focus on the relationships between immune cells and the intestinal microbiota, and research has revealed that the maturity of some immune cells is dependent on specific microbiota (e.g., some *Bacteroidetes* and *Firmicutes* species) [100]. Currently, the most studied intestinal immune cells are dendritic cells (DCs), macrophages, adaptive immune cells, and innate lymphoid cells (ILCs). Macrophages and DCs are the main antigen-presenting cells found under the IECs, which can identify both innocuous antigens and potential pathogens, ensuring that the host responds appropriately to the intestinal microbiota [101,102]. Adaptive immune cells are a type of immune cell that only participate in the adaptive immune response. They can undergo a complex process involving development, differentiation, maturation, and secretion after being stimulated by specific antigens. Key adaptive immune cells involved in the pathogenesis of IBD are T cells (including the T helper cells Th1, Th2, and Th17, and regulatory T [Treg] cells) [103]. ILCs are also an important class of immune cells that act as guards in the host protective immune system and also participate in immune-mediated diseases. It has been demonstrated that ILCs respond rapidly to intestinal ecosystem factors, such as luminal bacteria, metabolic signals, and cytokines [104]. It has also been demonstrated that some subsets are involved in the pathogenesis of IBD (NCR^-^ ILC3, ILC1). Additionally, some probably have protective functions (NCR^+^ ILC3) while others remain controversial (ILC2) [104,105]. Thus, the numerous intestinal immune cells have immune functions in the mucosal immune system (Figure 1).

## 4. Orchestrated Balance between Mucosal Immune System and Gut Microbiota

### 4.1. Interaction between Treg/Th17 Axis and Gut Microbiota

Tregs, Th1, Th2, and Th17 are all derived from the differentiation of naïve CD4^+^ T lymphocytes, which can be promoted by ILCs, DCs, and macrophages [106]. As one of the most studied CD4^+^ T helper cell subsets, Th17 cells are characterized by IL-17 production and secretion, which promotes intestinal inflammation [107]. Th17 cells are crucial for protecting the intestinal mucosal barrier from pathogens, comprising bacteria, fungi, and viruses [108]. However, in IBD patients, the majority of gut Th17 cells are found in ulcerative areas, and advanced mice experiments have also shown that abnormally elevated levels of Th17 cells (induced by specific bacteria) can exacerbate colitis [24].

Tregs are a subset of CD4^+^ T cells, and they are defined by the expression of CD25 and Foxp3 [109]. Tregs play a crucial role in the negative control of the immune system by producing IL-10 and transforming growth factor (TGF)-β, maintaining immune tolerance and immune homeostasis. Studies have shown that Treg defects and functional abnormalities are involved in the pathogenesis of various diseases, including IBD [110,111]. Therefore, the Treg/Th17 axis maintains the intestinal mucosal immune homeostasis and determines the incidence and severity of IBD. Correcting the imbalance of the Treg/Th17 axis may contribute to the alleviation of inflammation.

Since the establishment and application of germ-free mice, the relationship between the gut microbiota and the Treg/Th17 axis has been widely studied. Early studies showed that germ-free mice had fewer CD4^+^CD25^+^ T cells in mesenteric lymph nodes, suggesting that the gut microbiota favors the development of Treg cells [112]. Moreover, colonization with different types of microbiota or a single bacterial strain can trigger different immune responses and establish diverse gut immune landscapes [113]. In vitro co-culture experiments involving *Clostridium* and colon epithelial cells indicated that *Clostridium* induced TGF-β production, which promoted CD4^+^ T cell differentiation into Tregs [114]. A major species of the order Clostridia, *Faecalibacterium prausnitzii*, is one of the most abundant anaerobic intestinal bacteria. Research has confirmed that it promotes butyrate production and blocks the IL-6/Stat3/IL-17 pathway, thus reducing CD4^+^ T cell differentiation into Th17 cells and promoting Treg cells [115]. *Helicobacter pylori*, a pathogenetic Gram-negative bacterium, can cause gastric ulcers; however, research has indicated that it can ameliorate DSS-induced chronic colitis in mice, which may be associated with Th17 downregulation and Treg upregulation [116]. In brief, all these observations in mouse models supported the hypothesis that changes in the gut microbiota composition alter the balance of the Treg/Th17 axis, contributing to the aggravation or alleviation of IBD [117]. Subsequently, microbiota transfer from healthy or IBD donors to germ-free mice verified the correctness of this hypothesis [118].

**Figure 2:**
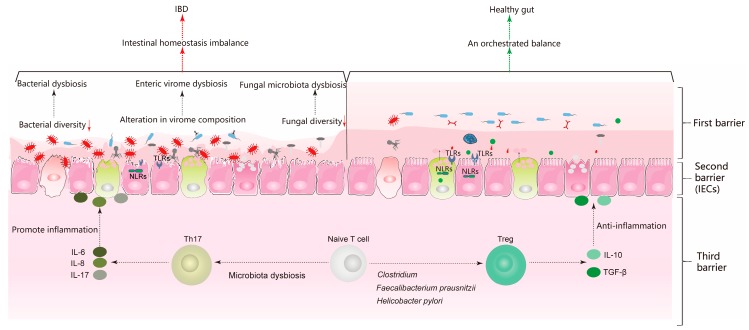
Alteration of the intestinal homeostatic balance promotes the pathogenesis of inflammatory bowel disease (IBD). During homeostasis in healthy intestines, gut microbes induce an immune tolerance phenotype. In contrast, the key features of homeostasis imbalance are microbiota dysbiosis and immunological dysregulation. Microbiota dysbiosis involves the excessive reproduction of potentially pathogenic microorganisms, which can erode the intestinal mucosa and increase intestinal permeability, thus promoting the overactivation of the adaptive and innate immune system and driving chronic inflammation. Moreover, microbiota dysbiosis can induce imbalance of the Treg/Th17 axis, leading to further inflammatory responses in the intestinal tissue. However, some gut microbiota (e.g., *Clostridium, Faecalibacterium prausnitzii*, and *Helicobacter pylori*) favors the development of Treg cells to promote the anti-inflammatory effect. In brief, any side or both abnormal in gut microbiota or Treg/Th17 axis may cause intestinal homeostatic imbalance. Ultimately, disorders of the intestinal homeostasis can lead to or aggravate IBD.

As a crucial transcription factor for maintaining the balance of the Treg/Th17 axis, c-Maf can regulate the differentiation and function of intestinal Treg cells. Research has shown that, in c-Maf-deficient mice, the intestinal microbiota was severely disturbed, and when transferred to germ-free mice, the microbiota induced severe intestinal Th17 responses and aggravated inflammatory reactions [119]. Moreover, the IL-17 receptor (IL-17R), which is a key IL-17 signaling pathway receptor responsible for driving Th17 cell development, is essential for regulating the effects of the mucosal immune system against intestinal pathogen infections and controlling gut microbiota dysbiosis [120]. As an indispensable subset of Treg cells, pTreg cells enriched in the intestines have a profound impact on intestinal microbial communities, and pTreg cell deficiency in mice induced pervasive changes in gut microbial metabolite profiles and the intestinal epithelium [121]. In brief, there is a sophisticated crosstalk between the Treg/Th17 axis and the gut microbiota. A Treg/Th17 axis imbalance can cause microbiota dysbiosis, and microbiota dysbiosis can also lead to the imbalance of the Treg/Th17 axis. Moreover, disorders of the Treg/Th17 axis or the gut microbiota can lead to or aggravate IBD (Figure 2).

### 4.2. Communication between Pattern Recognition Receptors (PRRs) and Gut Microbiota

**Figure 3:**
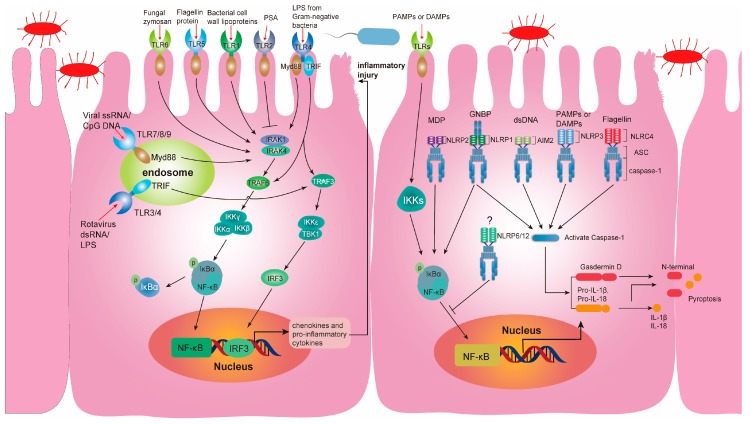
Diagram showing the activation pathways of toll-like receptors (TLRs) and NOD-like receptors (NLRs) in the intestinal epithelial cells. Intestinal epithelial cells (IECs) express multiple pattern recognition receptors (PRRs) including TLRs and NLRs, which can recognize pathogen-associated molecular patterns (PAMPs) and damage-associated molecular patterns (DAMPs). TLRs are present in cell membranes and endosomes. When TLRs sense PAMPs or DAMPs, they can recruit signaling adaptors (myeloid differentiation factor 88, MyD88) and then initiate a signaling cascade in MyD88 dependent mechanism, eventually causing the transcriptional activation of nuclear factor kappa-B (NF-κB). TLRs can also be activated in the MyD88 independent mechanism that involves TIR-domain-containing adaptor protein inducing interferon-β (TRIF). Canonical activation of NLR family pyrin domain-containing proteins (NLRPs) requires two signals. Signal 1 is activated by PAMPs or DAMPs through TLRs for the upregulation of pro-IL-18 and pro-IL-1β. Signal 2 involves the sensitization of NLRs and the assemble of inflammasome, which further induces the activation of caspase-1 to cleave pro-IL-18, pro-IL-1β, and Gasdermin D. Eventually, IL-18, IL-1β, and Gasdermin D N-terminal domain induce cell pyroptosis. TRAF: TNF receptor-associated factors; IRAK: IL-1R-associated kinases; IKK: inhibitor of NF-κB kinase; IκBα: inhibitor of NF-κBα; IRF: interferon-regulatory factors; TBK1: TANK-binding kinase 1; PSA: Polysaccharide A; ssRNA: single-stranded RNA; dsRNA: double-strand genomic RNA; CpG DNA: CpG-rich hypomethylated DNA motifs in microbial genome; GNBP: Gram-negative bacterial peptidoglycan; MDP: muramyl dipeptide.

PRRs are widely expressed in various cells of the intestinal mucosal immune system, including IECs, DCs, macrophages, adaptive immune cells, and ILCs. They are responsible for recognizing microorganisms’ different molecular patterns, thus preventing pathogen invasion and maintaining intestinal homeostasis [121,122,123,124,125]. A growing number of studies are finding that PRRs play a key role in both avoiding direct contact between gut microbiota and IECs and influencing the structure of intestinal communities [126]. At present, known PRRs include Toll-like receptors (TLRs), NOD domain-like receptors (NLRs), melanoma differentiation-associated gene 5 (*MDA5*), laboratory of genetics and physiology gene 2 (*LGP2*), and retinoid acid-inducible gene-I (*RIG-I*). However, among all the PRRs, TLRs and NLRs are the classical PRRs, and they have been widely studied and explored [127,128] (Figure 3).

### 4.3. TLRs and the Gut Microbiota

TLRs, the best-characterized transmembrane receptors, with at least 13 types, exist in various intestinal cells including IECs (e.g., Paneth cells and goblet cells) and resident immune cells in the intestinal lamina propria (e.g., macrophages and adaptive immune cells) [129]. Studies have confirmed that TLR1 to TLR9 all exist in IECs [130], but the exact mechanisms of TLRs underlying the regulation of intestinal homeostasis have yet to be fully illuminated. Bacterial cell walls lipoproteins, bacterial peptidoglycan, and fungal zymosan are recognized by TLR1, TLR2, and TLR6, respectively [131,132,133]. TLR4 is responsible for recognizing LPS produced by Gram-negative bacteria. TLR5 can recognize flagellin proteins, which are granular proteins constituting bacterial flagellum fiber [129].

Myeloid differentiation primary response gene 88 (MyD88) was identified as the TLR signaling pathway adaptor protein responsible for transmitting the TLR signal to downstream kinases [134]. MyD88 signaling regulates the production of certain AMPs in specialized IECs, maintaining the barrier functions of the intestinal epithelium [135]. MyD88-deficient mice cannot block pathogenic bacterial invasion into the intestinal epithelium [136].

TLR2 can recognize anti-inflammatory *Bacteroides fragilis* polysaccharide A (PSA) and initiate signaling to regulate the Treg/Th17 axis, thereby promoting immunologic tolerance [137]. PSA is only found in the human microbiome, and it activates an anti-inflammatory immune response that alleviates inflammatory disease [138]. However, the exact mechanism of TLR2 involvement in the development of IBD has not been fully elucidated because of paradoxical results in TLR2^-/-^ mice [139,140]. TLR5^-/-^ mice tend to develop colitis or systemic inflammation, and further research has shown that the mechanism is related closely to *E. coli* due to the altered gut microbiota composition in these mice [141]. Moreover, research has shown that genetic variants of TLR4 in the population lead to susceptibility to IBD [142]. However, TLR4 is highly expressed in colon segments where pathogenic bacterial invasion and infection are exacerbated in DSS-induced colitis [143]. As such, many studies have confirmed that TLRs communicate with the gut microbiota so as to mediate inflammatory immune responses and maintain intestinal epithelial homeostasis.

### 4.4. NLRs and the Gut Microbiota

NLRs expressed in the cytosol are essential for preventing the invasion of pathogenic bacteria. NLRs exist in various intestinal cells, including IECs and resident immune cells in the intestinal lamina propria [144,145]. At least 23 NLR proteins have been identified, but the mechanisms and biological functions of only a minority have been extensively studied [146,147]. NLRs are novel receptors that maintain intestinal epithelial homeostasis via communication and interaction with the gut microbiota. Remarkably, many NLR genes have been characterized as IBD susceptibility genes, as supported by several studies [148,149,150]. Some NLRs form multimolecular protein complexes, known as inflammasomes, with pro-caspase-1 and apoptosis-associated speck-like protein containing a CARD (ASC). These inflammasomes are assembled upon stimulation by damage-associated molecular patterns (DAMPs) or pathogen-associated molecular patterns (PAMPs). Furthermore, activated NLR inflammasomes can trigger caspase-1 activation and induce the production of mature IL-1β/IL-18, thereby provoking an immune response [151,152].

NOD1 can detect a unique γ-D-glutamyl-*meso*-diaminopimelic acid motif found predominantly in Gram-negative bacterial peptidoglycan, so as to initiate an inflammatory response [153]. NOD2 can recognize muramyl dipeptide (MDP) contained in peptidoglycan, which is found in Gram-positive and Gram-negative bacteria [154]. Exposure to MDP triggers a series of acute inflammatory signaling effects, inducing the production and secretion of inflammatory cytokines [155]. The most investigated NLR, NLR family pyrin domain-containing protein 3 (NLRP3), can be activated by various exogenous and endogenous ligands or stimuli, such as reactive oxygen species, ATP, bacteria, viruses, and fungi [156]. However, how NLRP3 maintains intestinal homeostasis remains controversial. Some studies show that NLRP3^-/-^ mice are prone to colitis, while other studies indicate that inflammatory reactions are reduced in these mice [157,158,159]. NLR family CARD domain-containing protein 4 (NLRC4) ligands include flagellin of bacteria such as *Salmonella* and PrgJ and CprI (subunits of bacterial type III secretion systems) [160,161,162]. NLRC4 protects the intestinal mucosal barrier by restricting intestinal pathogens such as *Citrobacter rodentium* and *Salmonella* [160,163]. The NLRP6 ligands remain unknown, but evidence has established a relationship between NLRP6 and the gut microbiota. Microbial genome sequencing has indicated that the gut microbiota is changed in NLRP6-deficient mice, with the levels of dominant bacteria (*Firmicutes, Bacteroidetes*, and *Proteobacteria*) being significantly altered [164,165]. Most interestingly, excessive NLRP12 activation inhibits NF-κB signal transduction. NLRP12^-/-^ mice also have the same characteristics, reflecting susceptibility to colitis and microbiome dysbiosis, which indicates the key role of NLRP12 in maintaining intestinal homeostasis [166].

## 5. Discussion and Conclusions

When intestinal homeostasis is maintained, the intestinal mucosal immune system can effectively resist pathogen invasion and inhibit excessive pathogen reproduction, and simultaneously, commensal intestinal bacteria maintain intestinal immune tolerance [167]. However, immune system–microbiota interactions act as a double-edged sword, with the microbiota being beneficial to the host in normal conditions, but also potentially causing adverse effects in the host that contribute to inflammation [168]. The gut microbiota is constantly monitored by the mucosal immune system, and any slight disturbance in the gut microbiota may contribute to intestinal immune disruption and increased susceptibility to IBD [169]. The intestinal mucosal immune system contains various signal transduction pathways that involve PRR signaling and adaptive T cell responses. PRRs are the first sensors of microorganisms (including pathogens, commensal bacteria, and conditional pathogens), and they act as part of the host defense system. However, it is not clear whether all microorganisms (such as bacteria, fungi, or viruses) are equally sensed by PRRs or whether there are more specific recognition and defense mechanisms for maintaining intestinal homeostasis. Moreover, although plenty of studies have shown the effects of pathogens and commensal bacteria on intestinal immune function, the influences of conditional pathogens on intestinal mucosal immune homeostasis are rarely reported. Nevertheless, a certain co-evolutionary relationship has been found between conditional pathogens and hosts, and conditional pathogens may activate the innate intestinal immune system, thus causing intestinal inflammation [106,170,171].

Interestingly, microorganism exposure in early life is crucial for the construction of the host immune system, and it helps the host to build early innate immune responses and regulate the development of autoimmune and inflammatory diseases such as IBD [5,167,172]. During the first few years of life, intestinal microorganisms can directly or indirectly affect the maturation of the intestinal mucosal immune system [173]. Moreover, *Clostridia* colonization of the neonatal intestinal tract contributes to the prevention of enteric pathogen growth [174]. Furthermore, by constantly monitoring pregnant mice and their offspring, a recent study showed that maternal microbial exposure during pregnancy shapes the intestinal immune system of the offspring, including the innate lymphoid and mononuclear cell populations [175]. The intestinal mucosal immunity (including the IEC functions, IgA production, and differentiation of T-cell subsets) of germ-free mice is very different from that of conventionally raised mice, and germ-free mice are more sensitive to DSS exposure [176,177,178,179].

Meanwhile, there is a potential link between PRR signaling deficiency (e.g., related to NOD2, MyD88, and TLR5) and the microbiota composition, and defects in certain PRRs may contribute to IBD susceptibility [136,141,180]. Remarkably, mutations of NOD2 loci in IBD patients are significantly correlated with compositional changes in the intestinal-associated microbiota, including increased *Escherichia* and decreased *Faecalibacterium* [181].

In summary, microbiota dysbiosis may affect the intestinal mucosal immune system and, in turn, immune system dysfunction may cause gut microbiota disorders. The mutual interaction between the intestinal immune system and the gut microbiota may contribute to the pathogenesis of IBD. However, evidence from research on this interactive relationship is still very limited, lacking the construction and comprehension of co-regulation network between signaling pathways and gut microbiota or its metabolites profiles. Adopting multidisciplinary and multi-domain technologies, combining with genomics, proteomics, metabonomics, rapidly maturing computer artificial intelligence and bioinformatics technology will be critical to further illuminate the perplexing mechanisms of gut microbiota-mucosal immune interactions in IBD. Accurately understanding and clarifying the complicated connections between gut microbiota and mucosal immune system, will help researchers to develop novel and effective therapies, and eventually cure IBD.

## Abbreviations

AIEC	adherent-invasive Escherichia coli
AMPs	antimicrobial peptides
ASCA	anti-Saccharomyces cerevisiae antibodies
ASC	apoptosis-associated speck-like protein containing a CARD
CD	Crohn’s disease
DAMPs	damage-associated molecular patterns
DCs	dendritic cells
DSS	dextran sulfate sodium
IBDs	inflammatory bowel diseases
ILCs	innate lymphoid cells
IECs	intestinal epithelial cells
LGP2	laboratory of genetics and physiology gene 2
LPS	lipopolysaccharides
MDA5	melanoma differentiation-associated gene 5
MUC2	mucin 2
MDP	muramyl dipeptide
MNV	murine norovirus
MyD88	myeloid differentiation primary response gene 88
NLRs	NOD domain-like receptors
NF-κB	nuclear factor kappa-B
PAMPs	pathogen-associated molecular patterns
PRRs	pattern recognition receptors
PSA	polysaccharide A
Treg	regulatory T
RIG-I	retinoid acid-inducible gene-I
SIgA	secretory immunoglobulin A
SCFA	short-chain fatty acids
TLRs	toll-like receptors
ILC2s	type 2 innate lymphoid cells
UC	ulcerative colitis
